# Enzyme characterization and biological activities of a resuscitation promoting factor from an oil degrading bacterium *Rhodococcus erythropolis* KB1

**DOI:** 10.7717/peerj.6951

**Published:** 2019-05-21

**Authors:** Dan Luo, Jixiang Chen, Gang Xie, Liang Yue, Yonggang Wang

**Affiliations:** 1 School of Petrochemical Engineering, Lanzhou University of Technology, Lan Zhou, China; 2 School of Life science and Engineering, Lanzhou University of Technology, Lan Zhou, China

**Keywords:** Resuscitation promoting factor, *Rhodococcus erythropolis*, VBNC, Muralytic activity, Resuscitation

## Abstract

Resuscitation-promoting factors (Rpf) are a class of muralytic enzymes, which participate in recovery of dormant cells and promoting bacteria growth in poor media. In the present study the expression vector of the *rpf-1* gene from an oil-degrading bacterium *Rhodococcus erythropolis* KB1 was constructed and expressed in *Escherichia coli.* The expressed protein was purified by Ni^2+^-affinity chromatography, and showed muralytic activity when measured with 4-methylumbelliferyl-β-D-N,N′,N″-triacetyl chitotrioside as substrate. Addition of purified Rpf-1 to *R. erythropolis* culture efficiently improved bacterial cell growth. The purified protein also increased resuscitation of viable but nonculturable cells of *R. erythropolis* to culturable state. The conserved amino acid residues including Asp^45^, Glu^51^, Cys^50^, Thr^60^, Gln^69^, Thr^74^, Trp^75^ and Cys^114^ of the Rpf-1 were replaced with different amino acids. The mutant proteins were also expressed and purified with Ni^2+^-affinity chromatography. The muralytic activities of the mutant proteins decreased to different extents when compared with that of the wild type Rpf-1. Gln^69^ was found to play the most important role in the enzyme activity, substitution of Gln^69^ with lysine (Q69K) resulted in the greatest decrease of muralytic activity. The other amino acid residues such as Asp^45^, Glu^51^, Cys^50^ and Cys^114^ were also found to be very important in maintaining muralytic activity and biological function of the Rpf-1. Our results indicated that Rpf-1 from *R. erythropolis* showed muralytic activities and weak protease activity, but the muralytic activity was responsible for its growth promotion and resuscitation activity.

## Introduction

Bacteria can adopt a dormant state when they are in harmful environments by reducing metabolic activity and ceasing to divide. Dormancy can take different forms, and some low G+C Gram-positive bacteria such as *Clostridium and Bacillus* have adopted sporulation as a means of surviving adverse environmental conditions (Rittershaus, Baek & Sassetti, 2013; Dworkin & Shah, 2010). The other bacteria, including some Gram-positive and most Gram-negative bacteria, enter a viable but nonculturable state (VBNC) in which the bacterial cells are viable as evidenced by their intact cell membranes and continued gene expression, but fail to replicate on routine laboratory media (Sachidanandham & Yew-Hoong Gin, 2009; Oliver, 2010). Upon encounter with favorable conditions, these dormant bacteria are activated and resumed growth, thereby becoming culturable (Ayrapetyan & Oliver, 2016).

Mukamolova et al. (2002b) found that a secreted protein named resuscitation-promoting factor (Rpf) from *Micrococcus luteus* culture can effectively promote recovery and growth of *Micrococcus luteus* and *Mycobacterium tuberculosis* cells in VBNC state. They reported that picomolar concentration of the protein was sufficient to convert dormant *Micrococcus* cells into actively growing cells (Mukamolova et al., 2002a, 2006). The Rpf of *Micrococcus luteus* was found to possess a c-type lysozyme like domain and show muralytic activity (Cohen-Gonsaud et al., 2004, 2005). The function of the Rpf was recognized as cleaving peptidoglycan. The *rpf* gene of *Micrococcus luteus* encoded 223 amino acid residues. A segment of 70 amino acids residue of the Rpf from *Micrococcus luteus* was known as the Rpf domain, in which a conserved glutamate and two highly conserved cysteine residues were found to be important in maintaining enzyme activity. The Rpf domain was both necessary and sufficient for biological activity. Besides the Rpf specific domain, a peptidoglycan attachment site (LysM domain) (Buist et al., 2008), secretory signal sequence or transmembrane helices were also found in the Rpf of *Micrococcus luteus*.

Genes resembling *Micrococcus luteus rpf* are widely spread among high G+C Gram-positive bacteria (Kell & Young, 2000; Ravagnani, Finan & Young, 2005). Some bacteria contain several representatives. *Mycobacterium tuberculosis* and *Mycobacterium bovis* contain five *rpf*-like genes (Kana et al., 2008; Garnier et al., 2003). *Streptomyces coelicolor* also encodes five Rpfs (Sexton et al., 2015). All of the Rpf proteins structurally possess the c-type lysozyme-like domain with a conserved glutamate in their active sites, but they share some functional redundancy. For example, the solution structure of RpfB of *Mycobacterium tuberculosis* showed a protein fold similar with both lysozyme and lytic transglycosidase. The RpfB was reported to cooperate with an endopeptidase RipA to cleave cell wall peptidoglycan and promote resuscitation of the dormant mycobacteria (Hett et al., 2007; Nikitushkin et al., 2015). They are also suggested to be the important virulence factors of *Mycobacterium tuberculosis* with roles in bacillary replication during acute disease and reactivation of chronic persistent infection (Rosser et al., 2017). Deletion all of the five *rpf* genes did not affect the viability of *Mycobacterium tuberculosis* cells, but the *rpf* deletion mutants lost their abilities to recovery from dormant state, and their abilities to initiate infections also decreased (Downing et al., 2005; Kondratieva et al., 2011). Homology studies of the enzymatic activity and interaction with other cell wall-modifying enzymes suggest that Rpf can modify peptidoglycan, but the mechanism of the Rpf-mediated recovery from dormancy remain to be fully elucidated.

The genus *Rhodococcus* is a diverse group of bacteria, which are distributed widely in natural environments. They are able to survive under extremely harsh conditions, and degrade a large number of organic compounds, including some persistant and toxic compounds, which make them potentially useful in environmental and industrial biotechnology (Van der Geize & Dijkhuize, 2004). They appear to have large genomes and harbor large linear plasmids. In addition, there is increasing evidence that multiple pathways and gene homologues are present, which further increase their catabolic versatility (Larkin, Kulakov & Allen, 2005). We have isolated an oil-degrading bacterium *Rhodococcus erythropolis* KB1 from oil contaminated soil. It could use different organic substrates as sole carbon sources. The strain grew well at a wide range of temperatures and NaCl concentrations, which suggested that the bacterium could be used for the soil bioremediation in severe environments (Yang et al., 2015). By comparing the genomic sequence, we have found four different *rpf* genes, among which *rpf-1* gene of 564 bp was cloned and its sequence was found to contain the *rpf* domain and share 28.24% of similarity with that of the *rpf C* gene of *Mycobacterium tuberculosis.* The gene of *rpf-2* is similar to the *rpf B* gene of *Mycobacterium tuberculosis*, in addition to the Rpf domain, there is a G5 superfamily protein domain and two DUF348 domains. *Rpf-3* gene is similar to the *rpf B* gene of *S. coelicolor* and contains a *rpf* domain, a G5 superfamily protein domain and three DUF348 domains, no significant signal peptide sequences were found. *Rpf-4* gene is similar to the *rpf A* gene of *S. coelicolor* and has a LysM domain in addition to the *rpf* domain. (Yue et al., 2018). In this paper we constructed an expression vector for the *rpf-1* gene of *R. erythropolis* KB1 and expressed it in *Escherichia coli.* The conserved residues of the *rpf-1* protein were respectively replaced and their enzyme and biological activities were analyzed. The muralytic activities and biological activities of the different mutant proteins were studied and compared with that of the wild-type protein. We found that Asp^45^, Glu^51^, Cys^50^, Thr^60^, Gln^69^, Thr^74^, Trp^75^and Cys^114^ of the Rpf-1 protein play vital roles in the enzyme activities and growth promoting effects. We also found that the muralytic activities were related to their growth promoting effects.

## Material and Methods

### Bacteria strains, plasmids and culture conditions

The bacteria strains and plasmids used in this work are listed in Table 1. *R. erythropolis* KB1 was isolated from oil contaminated soil in southwest China and identified by physiological and biochemical characteristics and 16S rDNA analysis (Yang et al., 2015). *R. erythropolis* KB1 and *E. coil* strains were grown on Luria-Bertani (LB) medium at 30 and 37 °C respectively. All chemical reagents were analytical grade.

**Table 1:** Bacteria strains and plasmids used in this study.

Strains or plasmids	Characteristics	Source
Strain		
*R. erythropolis* KB1	Wild-type	Laboratory collection
*E. coli* DH5α		TIANGEN, China
*E. coli* BL21 (DE3)		TIANGEN, China
BL21-pET-32a (+)-*rpf-1*	Amp^r^, BL21containing pET-32a (+)-*rpf-1*	This work
Plasmids		
pET-32a (+) vector	Amp^r^, 5.9 kb, high-copy-number cloning vector	Novagen
pUCm-T-*rpf-1*	Amp^r^, pUCm-T with an 564 bp fragment containing *rpf-1*	This work
pET-32a (+)-*rpf-1*	Amp^r^, pET-32a (+) with an 564 bp fragment containing *rpf-1*	This work

### Construction of expression vector containing the *rpf-1* gene of *R. erythropolis* KB1 and its purification

To construct the expression vector of the *rpf-1* gene of *R. erythropolis* KB1, a specific pair of primers (forward primer 5′-AAGCTTATGAGCGGACGCCATCGC-3′ with an *Hind*III site, reverse primer 5′-GAATTCCTTGAGGAACGTCTTCGC-3′ with a *EcoR*I site) was designed based on the sequences of *rpf-1* gene of *R. erythropolis* KB1. The pUCm-T vector containing the *rpf-1* gene of *R. erythropolis* KB1 was used as a template. The PCR reaction condition was as follows: 98 °C for 5 min, one cycle; 98 °C for 10 s, 55 °C for 15 s, 68 °C for 90 s, 35 cycles; with a final extension at 72 °C for 10 min. The PCR product was purified and digested with *Hind*III and *EcoR*I, it was then inserted into the pET-32a (+) vector (Novagen, Merck, Germany) digested with the same enzymes. The constructed ligation product pET-32a (+)-*rpf-1* was transformed into *E. coli* DH5α and then into *E. coli* BL21 (DE3).

The *E. coli* BL21 (DE3) containing the pET-32a (+)-*rpf-1* were inoculated into 100 mL LB medium supplemented with ampicillin antibiotics and cultivated at 37 °C overnight, it was then added to 1,800 mL of the same LB medium, continued to grow to an OD_595nm_ of 0.6 and were induced with 0.8 mmol/L isopropyl-β-D-thiogalactopyranoside (IPTG) at 25 °C for 3 h. The bacterial broth was placed in centrifuge tubes and centrifuged at 8,500×*g* for 20 min at 4 °C, and the bacterial cells were collected and re-suspended in 50 mL of lysis buffer (pH 8.0, 20 mM Tris–HCl, 10 mM imidazole, 500 mM NaCl). The mixture was then sonicated with Ultrasonic Processor (SCIENTZ-950E; Ningbo Xinzhi Biotechnology Co., Ltd, Ningbo, China) in an ice bath (power 240 W, work 8 s, interval 12 s, 99 cycles). The broken cell solution was then centrifuged at 8,500×*g* for 20 min at 4 °C. The supernatant was collected and filtered through a 0.22 μm sterile filter (Millipore, Burlington, MA, USA). The recombinant protein was purified with Ni^2+^-affinity chromatography (Novagen, Merck, Germany) according to the manufacturer’s instructions and analyzed by SDS-polyacrylamide gel.

### Mutation analysis of the conserved amino acid resides in the active site of the Rpf-1

The *rpf-1* gene sequence of *R. erythropolis* KB1 was analyzed and compared with those of the related bacteria. The hypothetical conserved amino acid resides for the enzymatic activities were selected and the encoding cordons corresponding to each of the conserved amino acid residues were substituted with those of the other amino acids. The sequences containing the site-directed mutagenesis were commercially synthesized by Sangon Biotech (Shanghai, Co., Ltd., Shanghai, China). Each mutation was confirmed by nucleotide sequence analysis. The sequences containing the site-directed mutagenesis were listed in Table 2. Then the mutant plasmids were transformed into *E. coli* BL21 (DE3). The mutant proteins were expressed and purified in the same way as described above.

**Table 2:** The conserved amino acid resides for substitution and the related oligo nucleotide sequences.

Mutant proteins	Mutations	Oligo nucleotides (5′–3′)
E51A	Glu^51^→Ala	GTCTCGCCCAGTGTGCGGCCGGCGGAAACTG
D45A+E51K	Asp^45^→Ala	CCGACTCCGACTGGTATCGTCTCGCCCAGTG
	Glu^51^→Lys	GTCTCGCCCAGTGTAAGGCCGGCGGAAACTG
C50G+C114T	Cys^50^→Gly	ATCGTCTCGCCCAGGGTGAGGCCGGCGGAAA
	Cys^114^→Thr	GCGCATGGCCGTCCACCTCCTCCAGCCTCGG
T60A	Thr^60^→Ala	ACTGTGCCATCAACGCGGGCAACGGTTACCA
Q69K	Gln^69^→Lys	ACCAGGGCGGACTGAAGTTCTCCCCCAGCAC
T74A	Thr^74^→Ala	AGTTCTCCCCCAGCGCGTGGAACGCACACGG
W75V	Trp^75^→Val	GTTCTCCCCCAGCACGGTGAACGCACACGGCGGCG

### Protein electrophoresis and Western blotting

SDS-PAGE on 12% acrylamide gels was performed as described by Laemmli (1970). After electrophoresis, proteins were transferred to a nitrocellulose membrane (Millipore, Burlington, MA, USA) using semidry western transfer apparatus (Bio-Rad Laboratories, Hercules, CA, USA). Western blot analysis was carried out using a rabbit anti-His taq antibody (1:1,000 dilution), followed by a horseradish peroxidase-conjugated goat anti-rabbit IgG. Dianilino benzene substrate was used for detection.

### Muralytic activity analysis of the purified recombinant proteins

To determine muralytic activity of the recombinant Rpfs, the artificial fluorogenic substrate, 4-methylumbelliferyl-β-D-N,N′,N″-triacetylchitotrioside (4-MUF-3-NAG) (Sigma, Neustadt an der Weinstraße, Germany) was used and the enzyme activity was analyzed as reported before (Mukamolova et al., 2006). Briefly, 100 μL of the recombinant protein (0.02 mg mL^−1^) was added to 300 μL of 22 μM Mcllvaine’s phosphate buffer (100 mM citric acid, 200 mM sodium phosphate, pH 5.2 containing 4-MUF-3-NAG). The mixture was then incubated at 37 °C for 30 min. Three mL of 0.3 M glycine/NaOH buffer (pH 10.6) was added to the mixture. The fluorescence intensity was detected with Varioskan LUX Multimode Microplate Reader (Thermo Fisher, Waltham, MA, USA) using excitation wavelength of 360 nm and a read-out of 455 nm. The result was compared with a standard curve of standard substance 4-methylumbelliferone. One unit of enzyme activity was defined as the amount of enzyme that generates 1.0 nmol of the product in 1.0 mL of reaction solution per minute.

To determine the effect of metal ions and chemical agents on enzyme activities of the recombinant Rpf-1, Mg^2+^, Ca^2+^, Zn^2+^, Co^2+^, dithiothreitol (DTT), phenylmethanesulfonyl fluoride (PMSF), ethylene diamine tetra acetic acid (EDTA) and ethylene glycol tetra acetic acid (EGTA) were added to the reaction mixtures respectively. The mixtures were then incubated at 37 °C for 30 min and the muralytic activities were determined.

### Protease activity of the purified recombinant Rpfs

Protease activity was determined with azocasein as substrate by the method of Kreger and Lockwood (Inamura, Nakai & Muroga, 1985). Reaction solution consisted of 0.5 mL of azocasein solution (five mg mL^−1^), 0.1 mL of the enzyme solution and 0.4 mL of DDW. The mixture was incubated for 20 min at 25 °C. Then 3.5 mL of 5% trichloroacetic acid was added to the mixture and centrifugated at 2,000×*g* for 5 min. The supernatant was collected and mixed with 4.5 mL of 0.5 M NaOH, the optical density was determined at 440 nm (OD_440_). One unit of protease activity was defined as the amount of enzyme producing an increase of 0.001 OD_440_ under the conditions.

### Effects of the purified recombinant Rpfs on the growth of *R. erythropolis* cells

*R. erythropolis* cells were grown in LB broth at 28 °C with shaking at 150 rpm overnight. The bacterial solution was then inoculated to 500 mL of LB broth and cultured at 28 °C. One mL of the bacterial culture was removed regularly at an interval of 4 h and the cell growth was monitored at a wavelength of 595 nm using an UV-2102PC UV-VIS spectrophotometer (Unico Instrument Co., Ltd., Shanghai, China). To determine the effect of the recombinant Rpfs on the growth of *R. erythropolis* KB1, the purified proteins were added to the bacterial solutions and cultured under the same conditions. The OD_595nm_ of different groups were detected.

### Effect of the purified recombinant Rpfs on the cultural abilities of *R. erythropolis* cells under cold and starvation conditions

The *R. erythropolis* cells were grown in LB broth at 28 °C overnight. The bacteria solution was harvested by centrifugation at 5,000 rpm at 4 °C for 10 min and washed three times with normal saline. The washed cells were then inoculated into 500 mL of steriled normal saline and maintained at 4 °C without shaking to induce the VBNC state. A total of 100 μL of the bacteria solution was removed regularly and the culturable cells were estimated by plating 0.1 mL of the samples on LB agar plates.

The recovery of the cold stressed cells was analyzed as reported before. Briefly, five mL of the bacteria solution was removed from the flask which had been kept at 4 °C for more than 1,350 days, and yeast extract was added to the solution at a final concentration of 0.025% (w/v). The bacteria cell mixture was then incubated at 28 °C and the culturable cell counts were estimated by plating 0.1 mL of the samples on LB agar. To determine effect of the recombinant Rpf-1 on the recovery of *R. erythropolis* cells from the stressed state, the purified recombinant Rpf-1 was added to the stressed cell samples. The mixtures were then incubated at 28 °C and the culturable cells were estimated by plate count method. All experiments were performed in triplicates.

## Results

### Expression and purification of the recombinant Rpf-1 of *R. erythropolis* KB1

**Figure 1:**
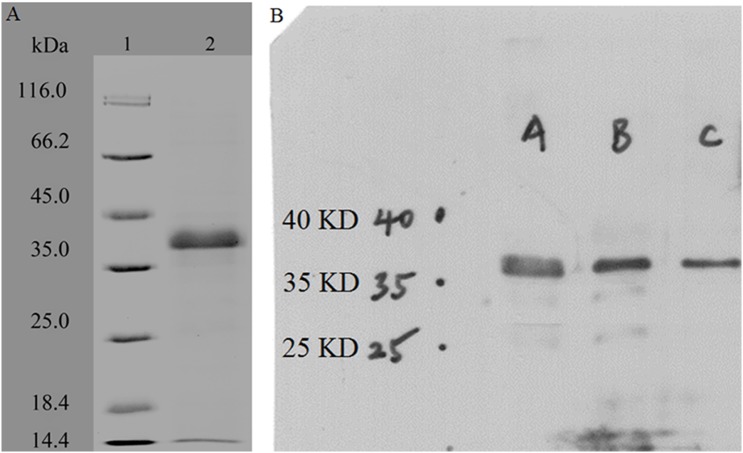
SDS-PAGE and Western blotting analysis of the purified recombinant Rpf-1. (A) SDS-PAGE analysis. (1) Protein molecular weight marker; (2) the purified Rpf-1; (B) Western blotting analysis with three replications.

The *rpf-1* gene of *R. erythropolis* KB1 consisted of 564 bp which encoded a polypeptide of 187 amino acids. The similarities with Rpf-1 of *Micrococcus luteus* and RpfC of *Mycobacterium tuberculosis* were 26.11% and 28.24% respectively. The polypeptide contained the Rpf domain of about 70 amino acids, in which a conserved glutamate (Glu^51^) and two highly conserved cysteine residues (Cys^50^ and Cys^114^) were found. But the LysM domain was not found, it did not contain signal sequence either. The *rpf-1* gene was inserted into pET-32a (+) to construct an expression vector pET-32a (+)-*rpf-1,* which was then transformed into *E. coli* BL21 (DE3) and expressed by introducing with IPTG at 25 °C. The recombinant Rpf-1 was purified by Ni^2+^-affinity chromatography from the whole-cell lysate preparation. The purified recombinant protein showed a single band with a mass of 37 kDa on SDS-PAGE, which corresponded to the deduced molecular weight of the Rpf-1 (18.80 kDa) and the fusion protein Trx-His-S tag of pET-32a (+) (Fig. 1A). The Western blotting analysis of the purified recombinant protein also showed a specific band of approximately 37 kDa (Fig. 1B). The recombinant Rpf-1 showed muralytic activity when determined with the artificial lysozyme substrate 4-methylumbelliferyl-β-D-N,N′,N″-triacetyl chitotrioside, with a specific activity of 1,760 units mg^−1^. The muralytic activity was not affected with 0.1 mM of Ca^2+^, Zn^2+^ and Co^2+^, DTT, EDTA, EGTA and serine protease inhibitor PMSF, but 0.1 mM of Zn^2+^ increased the enzyme activity by almost two fold (Table 3).

**Table 3:** Effects of metal ions and chemical agents on activities of the purified recombinant Rpf-1 of *R. erythropolis* KB1.

	Chemical reagents	Final concentrations (mM)	Relative activity (%)
Control			100.00
	Zn^2+^	0.1	260.83 ± 12.25
	Mg^2+^	0.1	96.90 ± 3.98
	Co^2+^	0.1	98.40 ± 2.55
	Ca^2+^	0.1	110.14 ± 3.59
	DTT	0.1	98.88 ± 4.21
	PMSF	0.1	109.43 ± 5.07
	EDTA	0.1	99.81 ± 3.22
	EGTA	0.1	98.49 ± 4.14

**Note:**

Purified enzyme was pre-incubated with metal ions and biochemical reagents for 30 min. The enzyme activities of the pre-incubated recombinant protein without reagents were taken as 100%. The activities represent the mean of at least two determinations carried out in duplicates, and the value display with the mean ± SD.

### Expression and purification of the mutant proteins

**Figure 2:**
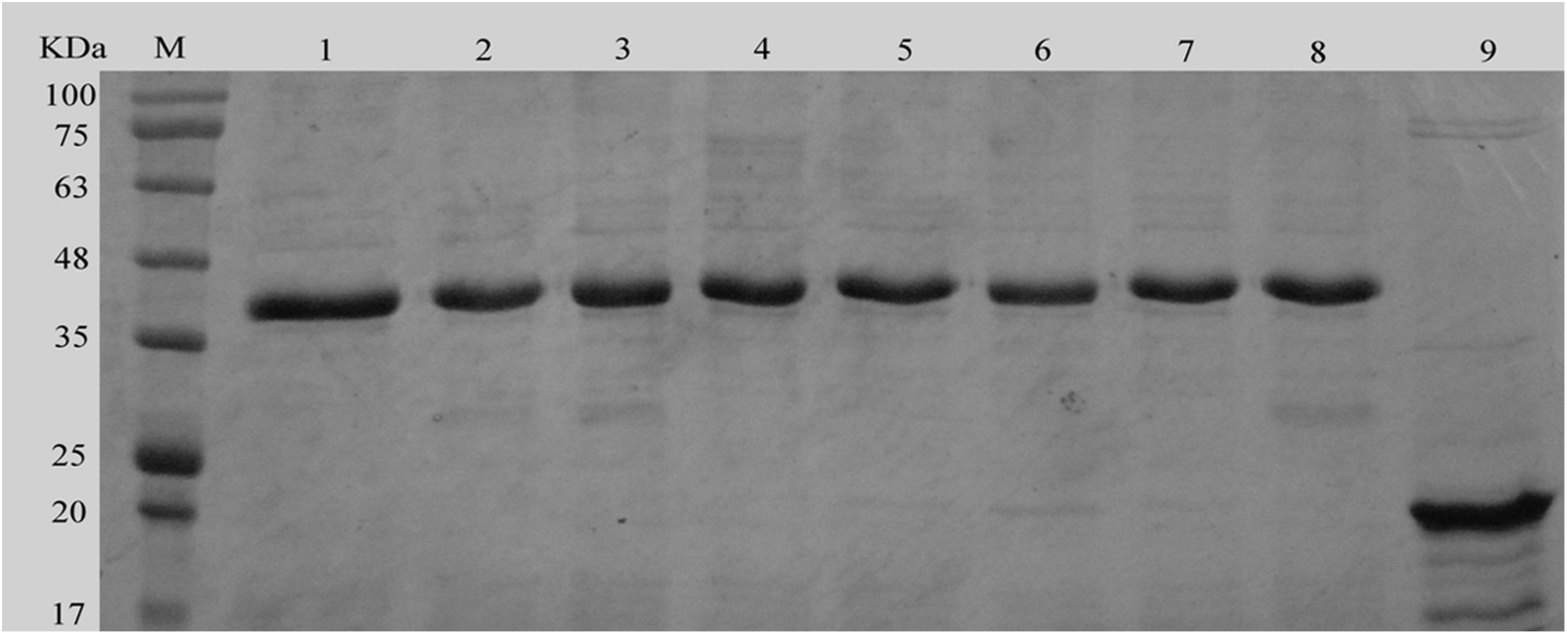
SDS-PAGE analysis of the mutant and wild-type Rpfs. M protein markers; one to seven the purified recombinant Rpfs containing with the different mutations of the conserved amino acid residues of E51A, C50G+C114T, D45A+E51K, T60A, Q69K, and T74A; eight wild-type Rpf-1; 9: pET-32a (+).

Based on the sequence alignments with Rpfs of the other bacteria, including the Rpf of *Micrococcus luteus*, RpfA-E of *Mycobacterium tuberculosis* and of *S. coelicolor,* and lysozyme C from animals, a total of seven highly conserved amino acid residues of the Rpf-1 of *R. erythropolis* KB1 were selected and substituted with other amino acids. The mutant proteins were expressed in *E. coli* BL21 (DE3) and purified with Ni^2+^-affinity chromatography. The purified mutant Rpfs showed single bands with the molecular weight of approximately 37 kDa on SDS-PAGE (Fig. 2), which were as the same size with that of the wild-type Rpf-1, but the muralytic activities of the mutant proteins varied among the different substitutions of amino acid residues (Table 4). For example, substitution of the conserved glutamate in position 51 with alanine (E51A) resulted in partial loss of activity (11.14%). But substitution of the aspartic acid in position 45 with lysine and the glutamate 51 with alanine (D45A+E51K) essentially suppressed the enzymatic activities (84.43%). Substitution of the two cysteines Cys^50^ and Cys^114^ (C50G+C114T) inhibited maximally the activities (86.68%). When the glutamine in position 69 was substituted with lysine (Q69K) the activity was greatly decreased (86.11%). However, the substitution of the tryptophan in position 75 with valine (W75V) increased the activity by almost two times.

**Table 4:** The enzymatic activities of the wild-type and mutant Rpfs of *R. erythropolis* KB1.

Proteins	Muralytic activity		Protease activity	
	Specific activity (u mg^−1^)	Percent activity retained (%)	Specific activity (u mg^−1^)	Percent activity retained (%)
Rpf-1	1,760 ± 129	100.00	1,630 ± 310	100.00
E51A	1,570 ± 230	88.80 ± 6.60	0	0
D45A+E51K	270 ± 30	15.60 ± 0.60	0	0
C50G+C114T	230 ± 10	13.30 ± 0.40	0	0
T60A	1,700 ± 100	96.70 ± 1.00	1,560 ± 100	98.30 ± 12.80
Q69K	240 ± 20	13.90 ± 0.30	0	0
T74A	1,700 ± 80	96.70 ± 2.20	0	0
W75V	3,430 ± 420	194.40 ± 9.70	0	0

**Note:**

The activities were determined at three replications and display with the mean ± SD.

### Effects of the recombinant Rpfs on the growth of *R. erythropolis* KB1

**Figure 3:**
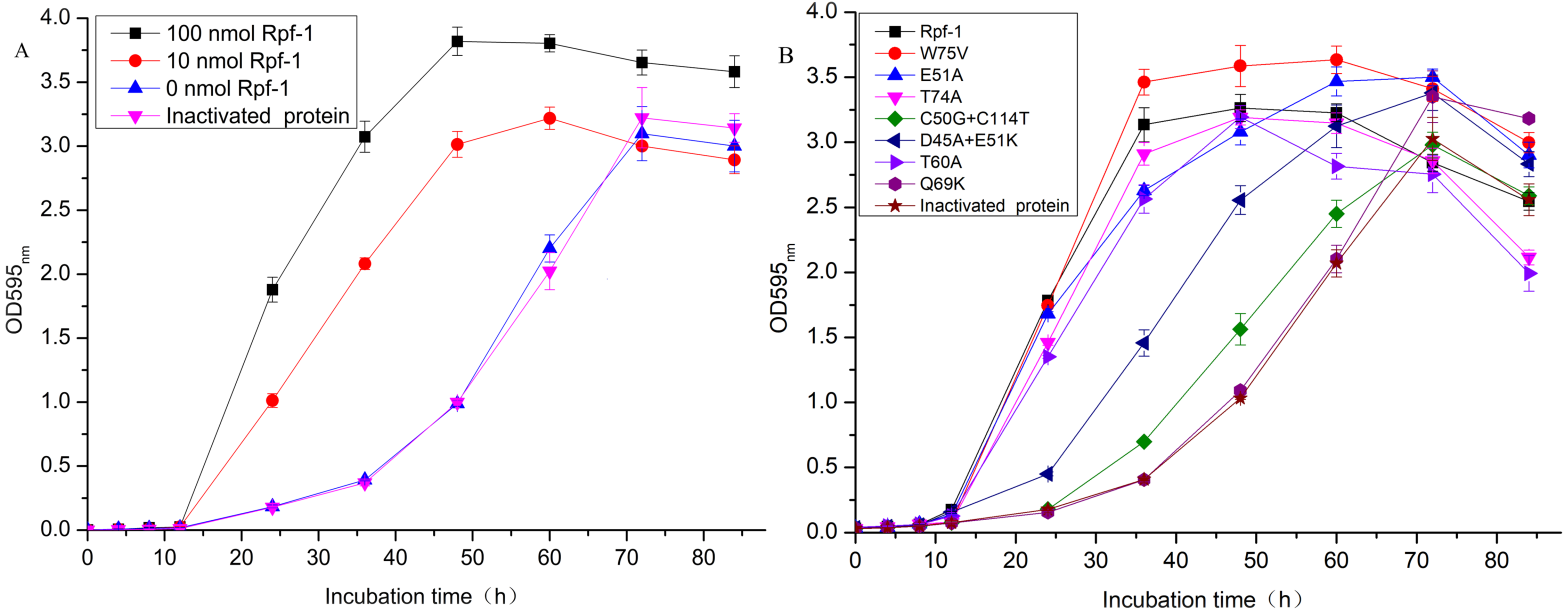
Growth curves of the *R. erythropolis* cells in LB medium with addition of different amount of wild-type proteins (A) and different mutant proteins (B).

The purified recombinant Rpfs of both wild-type and mutant proteins were added to the *R. erythropolis* KB1 cultures to determine the effect on the growth of the bacteria cells. As seen in Fig. 3, addition of the purified recombinant Rpf-1 efficiently stimulated the growth and obviously reduced the logarithmic phase of *R. erythropolis* KB1 cells from 24 to 12 h. The growth rate reached the maximum in 48 h, and the promoting effect was correlated with the amount of the Rpf-1. The growth abilities were increased for 301.70% and 382.28% respectively in 48 h by addition of 10 nmol and 100 nmol of the recombinant Rpf-1. The biological activities of the Rpfs were found to be correlated with its muralytic activities. Addition of the recombinant Rpf-1 mutant with W75V increases the growth more effectively when compared with that of the equal amount of the wild type Rpf-1. The growth rates of the bacterial cells added with the wild-type Rpf-1 and mutant Rpf with W75V were 316.18% and 347.22% respectively when compared with that of the control group, with the maximum growths in 48 h. The growth level of the mutant Rpf with E51A group decreased slightly when compared with that of the wild-type group. Addition of the Rpf mutants with the C50G+C114T and Q69K showed less effects on the cell growth (from 105.72–151.45%), with the log phases of about 24 h. The growth rates of the mutants with C50G+C114T, D45A+E51K and the control groups reached to the maximum in 72 h (Table 5).

**Table 5:** The growth ability of the *R. erythropolis* cells with addition of different mutant proteins.

Proteins	OD595_nm_ at 48 h	Relative ability (%)	logarithmic time (h)
Inactivated protein (control group)	1.032 ± 0.057	100.00	24
Wild type Rpf-1	3.263 ± 0.101	316.18 ± 7.70	12
E51A	3.079 ± 0.087	298.35 ± 8.08	12
D45A+E51K	2.556 ± 0.125	247.67 ± 1.57	24
C50G+C114T	1.563 ± 0.033	151.45 ± 5.18	24
T60A	3.194 ± 0.317	309.50 ± 13.66	12
Q69K	1.091 ± 0.163	105.72 ± 9.98	24
T74A	3.192 ± 0.211	309.11 ± 3.37	12
W75V	3.587 ± 0.265	347.22 ± 6.50	12

**Note:**

The growth ability was determined at three replications and display with the mean ± SD.

### Recovery of the *R. erythropolis* KB1 cells from cold and starved condition by addition of the recombinant Rpfs

**Figure 4:**
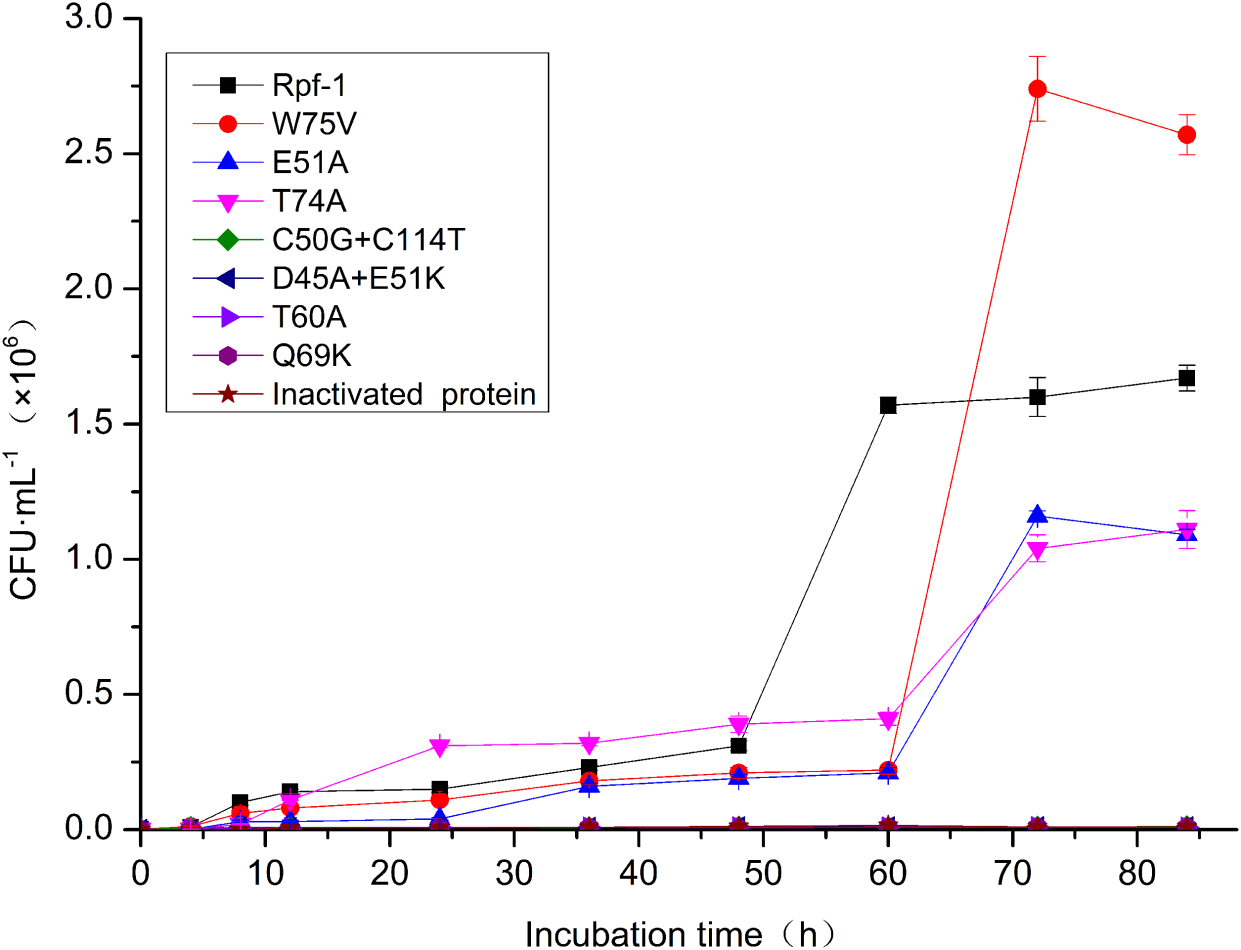
Recovery curves of *R. erythropolis* KB1 cells under cold stressed condition by addition of the different mutant Rpfs.

The *R. erythropolis* cells were inoculated into 500 mL of sterile normal saline and maintained at 4 °C without shaking for more than 1,350 days to induce the VBNC state. The stressed cells were then determined for their recover abilities at 28 °C with yeast extract. The effects of recombinant Rpfs on recovery of the *R. erythropolis* cells from the stressed state were also analyzed. As shown in Table 6 and Fig. 4, the culturable cells of *R. erythropolis* were decreased from 2.37 × 10^7^ to 8.50 × 10^3^ CFU mL^−1^ when they were maintained at 4 °C for more than 1,350 days, with 99.96% of the bacteria cells becoming unculturable. Addition of recombinant Rpf-1 noticeably increased the culturable counts of the cold stressed bacteria from 8.50 × 10^3^ to 1.67 × 10^6^ CFU mL^−1^. Addition of the recombinant mutant Rpf with W75V also increased the recovery more effectively when compared with that of the equal amount of the wild type Rpf-1. The mutant Rpfs with E51A and T74A increased the recovery of the bacteria cells at certain degrees, while the mutant Rpfs with D45A+E51K, C50G+C114T, Q69K and T60A did not show obvious promoting effect on the stressed bacterial cells.

**Table 6:** Recovery of the *R. erythropolis* KB1 cells under cold and starved condition by addition of the recombinant Rpfs.

Proteins	Culturable counts CFU mL^−1^ (×10^3^)	Percent recovery retained (%)
Inactivated protein	8.50 ± 0.47	100.00
Rpf-1	1,671.28 ± 52.69	19,688.04 ± 468.75
E51A	1,163.74 ± 19.21	13,720.51 ± 532.66
D45A+E51K	8.80 ± 0.32	103.63 ± 1.96
C50G+C114T	9.40 ± 0.92	110.57 ± 4.48
T60A	10.20 ± 0.98	119.66 ± 4.84
Q69K	8.20 ± 1.02	96.10 ± 7.31
T74A	1,120.02 ± 76.39	13,167.27 ± 170.63
W75V	2,742.15 ± 125.07	32,277.73 ± 313.18

**Note:**

Values are the means of three replication ± SD.

## Discussion

The Rpf proteins are widely distributed in Gram positive bacteria of the Actinobacteria phylum (Finn et al., 2008). The bacteria of *Rhodococcus* genus belong to the same order as *Micrococcus luteus*, Actinomycetales. Four *rpf* genes were found in *R. erythropolis* KB1, among which the *rpf-1* was one of the small genes with 564 bp, encoding 187 amino acids. The sequence of the Rpf-1 showed 26.11%, 28.22% and 32.24% similarities with Rpf of *Micrococcus luteus*, RpfC of *Mycobacterium tuberculosis* and RpfA of *S. coelicolor* respectively. The protein contained only an Rpf domain of about 70 amino acids. A conservative glutamic acid residue in the active center of the lysozyme-like proteins was found in the position 51 (Glu^51^), which was in the position 54 of the Rpf protein from *Micrococcus luteus*. The two highly conserved cysteine residues (Cys^50^ and Cys^114^) were also found in the Rpf-1 protein. The catalytic aspartic acid residues (Asp^70^) of the C-type lysozyme (*Gallus gallus*) and the catalytic threonine residue (Thr^50^) of the lysozyme of *E. coli* (Chauviac et al., 2014) were conserved in the Rpf protein (Asp^45^ and Thr^60^). The purified recombinant protein showed a 37 kDa band on SDS-PAGE, which corresponded to the deduced molecular weight of the Rpf-1 and the fusion protein Trx-His-S tag of pET-32a (+). The purified recombinant protein showed muralytic activity. The muralytic activity was not affected with 0.1 mM of Ca^2+^, Mg^2+^ and Co^2+^, DTT, EDTA, EGTA and serine protease inhibitor PMSF, but 0.1 mM of Zn^2+^ increased the enzyme activity by almost two times. In order to confirm the muralytic activity was produced by the recombinant Rpf-1 protein, we also purified and detected the enzyme activities of the purified Trx-His-S tag protein expressed by the empty vector pET-32a (+) under the same conditions, which showed no muralytic activity and biological activities (Dataset S3).

Mukamolova et al. (2006) reported that substitution of the hypothetical catalytic glutamate in Rpf of *Micrococcus luteus* by alanine (E54A) or lysine (E54K) essentially suppressed the enzymatic activity. Substitution of either Cys^53^ or Cys^114^ separately resulted only in partial loss of the activity, but substitution of both amino acids caused virtually complete inactivation of Rpf. The disulfide bond formation between the conservative cysteine residues of RpfC of *Mycobacterium tuberculosis* was recognized to support and modulate the catalytic domain conformation during catalysis by using NMR analysis (Maione et al., 2015). In GTP cyclohydrolase I, the thiol groups of two cysteine residues are involved in the chelation of Zn^2+^ in the active center of the enzyme. Addition of mercaptoethanol or DTT in renaturation buffer did not abolish Rpf activity, suggesting that disulphide bridge formation is not essential for the muralytic activity of the Rpf (Mukamolova et al., 2006).

Our results also showed that substitution of the two cysteines Cys^50^ and Cys^114^ (C50G+C114T) inhibited maximally the activities, and replacement both of the Asp^45^and Glu^51^ (D45A+E51K) greatly suppressed the enzymatic activities, which was consisted with the Rpf of *Micrococcus luteus.* But substitution of the conserved Glu^51^ with alanine (E51A) resulted only in partial loss of activity. According to the modeling and the structural data, residue Gln^72^ would be accessible within the active site cleft and might potentially be involved in the interaction of Rpf with its substrate. The Q72K replacement reduced muralytic activity against *Micrococcus luteus* cell walls by about 50.00%. But the muralytic and physiological activities of a protein bearing both Q72K and E54K alterations were not more significantly attenuated than those of the E54K mutant. We found that the Gln^69^ play very important roles and substitution of Gln^69^ with lysine (Q69K) resulted in the great decrease of the muralytic activity. Substitution of Trp^60^ with valine (W60V) had little effect on the enzymatic activity. While substitution of Trp^75^ with valine (W75V) increased the activity by almost two times, which showed the very important roles of this amino acid in enzymatic activities.

The purified recombinant Rpf-1 could efficiently stimulate the growth of *R. erythropolis* KB1 and reduced the logarithmic phase from 24 to 12 h. And the mutant Rpfs have different growth promoting effects on *R. erythropolis* KB1. The promoting effect was showed to be correlated with amounts of the Rpf-1. The biological activities of the Rpf-1 were also found to be correlated with its muralytic activities. The mutant Rpfs with low muralytic activities also decreased growth promoting effects and recovery of the bacteria cells from VBNC state. For example, the biological function of the mutant Rpf with substitutions of Cys^50^ and Cys^114^ (C50G+C114T) was greatly reduced when it was added to the *R. erythropolis* KB1 cells. The highest growth promoting effect was founded in the wild type Rpf-1 and the mutant proteins with Trp^75^, while the lowest promoting effects were founded among the substitutions of Asp^45^+Glu^51^ and Cys^50^+Cys^114^.

The purified recombinant Rpf-1 also showed weak protease activities when determined with azocasein. Substitutions of Glu^51^, Gln^69^, Thr^74^, Trp^75^, Asp^45^+Glu^51^, Cys^50^+Cys^114^ with other amino acids lost their protease activities. Only the mutant protein with Thr^60^ retained 95.90% of the protease activity of the wild-type protein. The protease activity did not show obvious correlation with the growth promoting and recovery activities. The mutant of Thr^74^ still retained the most growth promoting effect, although its protease activity was lost completely. It has been reported that the RpfB of *Mycobacterium tuberculosis* had a partner RipA (Resuscitation promoting factor interacting protein), which was a proteolytic enzyme capable of cleaving peptide bonds within the peptide chain of peptidoglycan. RipA is supposed to play an important role during the final stage of the cell division (Hett et al., 2007). Since RpfB can hydrolyze the glycoside bond between the residues of N-acetylglucosamine and Nacetyl muramic acid, while RipA is active at the D-Glu-meso-DAP sites of the peptide chain of the peptidoglycan, their interaction should result in a synergistic hydrolysis of the bacterial cell wall. It has also been experimentally shown that the combined effect of RpfB and RipA proteins on the hydrolysis of the fluorescently labeled peptidoglycan was stronger than the effects of individual RpfB and RipA proteins (Squeglia et al., 2014). Similarly, addition of both proteins also demonstrated synergistic effect in the procedure of reactivation of the dormant *Mycobacterium smegmatis* cells. The function of the protease activity of Rpf of *R. erythropolis* KB1 remains to be studied further.

Our results from *R. erythropolis* also showed that muralytic activity of the Rpf-1 was greatly related to its physiological function. But the precisely mechanism was not unclear. Rpf of *Micrococcus luteus* was initially assigned to the group of bacterial cytokines due to its activity in picomolar concentration (Mukamolova et al., 1998; Hett et al., 2008), but relatively high concentration of Rpf in the cultural medium of *Micrococcus luteus* and the absence of hypothetical receptors on the cell surface failed to confirm this initial hypothesis. Lytic transglycosylases may be important for the transport of macromolecules across the bacterial cell wall (Koraimann, 2003). It has been hypothesized that Rpf could facilitate the penetration of some compounds required for the resuscitation or growth of the bacteria. It is also possible that resuscitation and growth stimulation are indirect manifestations of the hydrolytic activity of Rpf. Nikitushkin proposed that the enzymatic hydrolysis of peptidoglycan under the influence of Rpf could be important for stimulation of growth and resuscitation of the dormant mycobacteria. They suggested three hypotheses for mechanism of action of the Rpf protein (Nikitushkin, Demina & Kaprelyants, 2016). The Rpf may act as a hydrolase to modify the peptidoglycan in the dormant cells and promote synthesis and growth of the cell wall, and stimulate the beginning of division process in the VBNC cells, because the dormant *Micrococcus luteus* cells have a significantly thickened cell wall. The second hypothesis is based on the ability of recombinant Rpf to disperse bacterial aggregations, which seems due to its hydrolytic activity (Voloshin & Kapreliants, 2005). It was found that aggregation of *Micrococcus luteus* cells was important for initiation of the growth of the cells at lag-phase as well as during the initial steps of reactivation of *Mycobacterium tuberculosis.* The Rpf proteins can participate in dispersion of these aggregates before the beginning of cell division. The third, Rpf can release low molecular weight molecules transmitting the signal onto the neighboring cells and acting on a surface cellular receptor. Muropeptides are considered as potential signaling molecules involved in organization of molecular parasite-host cascades (Nikitushkin, Demina & Kaprelyants, 2011). Disintegration of bacterial aggregations under the influence of Rpf can precede the subsequent generation of muropeptides triggering the resuscitation (Shleeva et al., 2003; Boneca, 2005). Because the resuscitation of the dormant bacterial forms is a complex phenomenon and includes several stages, whether the mechanisms of the resuscitation promoting of the dormant cells is same with that of the growth promoting of the normal cells remained to be discovered.

## Conclusion

We constructed the recombinant plasmid BL21-pET-32(a)-*rpf-1* of the *rpf-1* gene of *Rhodococcus erythropolis* KB1, and used the site-directed mutagenesis technique to *rpf-1* gene sequence muralytic activity related amino acids Asp^45^, Cys^50^, Glu^51^, Thr^60^, Gln^69^, Thr^74^, Trp^75^ and Cys^114^, several different Rpf amino acid mutant plasmids were constructed, and they were efficiently expressed and purified in *E. coli*. The molecular weight of the expressed protein was 37 kDa by SDS-PAGE electrophoresis.

The muralytic activity of the recombinant Rpf protein was 1,760 U/mg and the protease activity was 1,634 U/mg. 0.1 mM Zn^2+^ can increase muralytic activity, but Ca^2+^, Mg^2+^, Co^2+^, DTT, EDTA, PMSF and so on have little effect on muralytic activity. The purified recombinant Rpf protein promoted the growth of *R. erythropolis* KB1 and promoted the resuscitation of VBNC state KB1 cells. The resuscitation effect was more significant with the increase of recombinant Rpf protein concentration.

It was found that the muralytic activity of Rpf-1 changed after mutation, and the muralytic activity of C50G+C114T mutant lost by 86.68%, D45A+E51K mutant lost by 84.43%, Q69K mutant lost by 86.11%, and E51A mutant lost by 11.14%. While the muralytic activity of the W75V mutant increased by 94.45%. The growth-promoting and revitalizing effects of the C50G+C114T mutant disappeared, and the W75V mutant significantly increased the growth of *R. erythropolis* cells and the recovery of non-culturable cells. The growth-promoting and resuscitation effects of Rpf are closely related to its muralytic activity.

## Supplemental Information

10.7717/peerj.6951/supp-1 Supplemental Information 1 *rpf-1* sequence.

10.7717/peerj.6951/supp-2 Supplemental Information 2 The original data for enzyme activity determination.

10.7717/peerj.6951/supp-3 Supplemental Information 3 pET-32(a) protein.

## Additional Information and Declarations

### DNA Deposition

The following information was supplied regarding the deposition of DNA sequences: The rpf-1 data is available in a Supplemental File.

### Data Availability

The following information was supplied regarding data availability: Raw data is available in the Supplemental Files.

## References

[ref-1] Ayrapetyan M, Oliver JD (2016). The viable but non-culturable state and its relevance in food safety. Current Opinion in Food Science.

[ref-2] Boneca IG (2005). The role of peptidoglycan in pathogenesis. Current Opinion in Microbiology.

[ref-3] Buist G, Steen A, Kok J, Kuipers OR (2008). LysM, a widely distributed protein motif for binding to (peptido)glycans. Molecular Microbiology.

[ref-4] Chauviac FX, Robertson G, Quay DH, Bagnéris C, Dumas C, Henderson B, Ward J, Keep NH, Cohen-Gonsaud M (2014). The RpfC (Rv1884) atomic structure shows high structural conservation within the resuscitation-promoting factor catalytic domain. Acta Crystallographica Section F Structural Biology Communications.

[ref-5] Cohen-Gonsaud M, Barthe P, Bagnéris C, Henderson B, Ward J, Roumestand C, Keep NH (2005). The structure of a resuscitation-promoting factor domain from *Mycobacterium tuberculosis* shows homology to lysozymes. Nature Structural & Molecular Biology.

[ref-6] Cohen-Gonsaud M, Keep NH, Davies AP, Ward J, Henderson B, Labesse G (2004). Resuscitation-promoting factors possess a lysozyme-like domain. Trends in Biochemical Sciences.

[ref-7] Downing KJ, Mischenko VV, Shleeva MO, Young DI, Young M, Kaprelyants AS, Apt AS, Mizrahi V (2005). Mutants of *Mycobacterium tuberculosis* lacking three of the five rpf-like genes are defective for growth in vivo and for resuscitation in vitro. Infection and Immunity.

[ref-8] Dworkin J, Shah IM (2010). Exit from dormancy in microbial organisms. Nature Reviews Microbiology.

[ref-9] Finn RD, Tate J, Mistry J, Coggill P, Eberhardt RY, Eddy SR, Heger A, Hetherington K, Holm L, Mistry J, Sonnhammer EL, Tate J, Punta M (2008). Pfam: the protein families database. Nucleic Acids Research.

[ref-10] Garnier T, Eiglmeier K, Camus JC, Medina N, Mansoor H, Pryor M, Duthoy S, Grondin S, Lacroix C, Monsempe C, Simon S, Harris B, Atkin R, Doggett J, Mayes R, Keating L, Wheeler PR, Parkhill J, Barrell BG, Cole ST, Gordon SV, Hewinson RG (2003). The complete genome sequence of *Mycobacterium bovis*. Proceedings of the National Academy of Sciences of the United States of America.

[ref-12] Hett EC, Chao MC, Deng LL, Rubin EJ (2008). A mycobacterial enzyme essential for cell division synergizes with resuscitation-promoting factor. PLOS Pathogens.

[ref-13] Hett EC, Chao MC, Steyn AJ, Fortune SM, Deng LL, Rubin EJ (2007). A partner for the resuscitation promoting factors of *Mycobacterium tuberculosis*. Molecular Microbiology.

[ref-14] Inamura H, Nakai T, Muroga K (1985). An extracellular protease produced by *Vibrio anguillarum*. Bulletin of the Japanese Society for the Science of Fish.

[ref-15] Kana BD, Gordhan BG, Downing KJ, Sung N, Vostroktunova G, Machowski EE, Tsenova L, Young M, Kaprelyants A, Kaplan G, Mizrahi V (2008). The resuscitation-promoting factors of *Mycobacterium tuberculosis* are required for virulence and resuscitation from dormancy but are collectively dispensable for growth in vitro. Molecular Microbiology.

[ref-16] Kell DB, Young M (2000). Bacterial dormancy and culturability: the role of autocrine growth factors. Current Opinion in Microbiology.

[ref-17] Kondratieva T, Rubakova E, Kana BD, Biketov S, Potapov V, Kaprelyants A, Apt A (2011). *Mycobacterium tuberculosis* attenuated by multiple deletions of rpf genes effectively protects mice against TB infection. Tuberculosis (Edinb).

[ref-18] Koraimann G (2003). Lytic transglycosylases in macromolecular transport systems of Gram-negative bacteria. Cellular and Molecular Life Sciences (CMLS).

[ref-19] Laemmli UK (1970). Cleavage of structural proteins during the assembly of the head of bacteriophage T4. Nature.

[ref-20] Larkin MJ, Kulakov LA, Allen CCR (2005). Biodegradation and *Rhodococcus*–masters of catabolic versatility. Current Opinion in Biotechnology.

[ref-21] Maione V, Ruggiero A, Russo L, De Simone A, Pedone PV, Malgieri G, Berisio R, Isernia C (2015). NMR structure and dynamics of the resuscitation promoting factor RpfC catalytic domain. PLOS ONE.

[ref-22] Mukamolova GV, Kaprelyants AS, Young DI, Young M, Kell DB (1998). A bacterial cytokine. Proceedings of the National Academy of Sciences of the United States of America.

[ref-23] Mukamolova GV, Murzin AG, Salina EG, Demina GR, Kell DB, Kaprelyants AS, Young M (2006). Muralytic activity of *Micrococcus luteus* Rpf and its relationship to physiological activity in promoting bacterial growth and resuscitation. Molecular Microbiology.

[ref-24] Mukamolova GV, Turapov OA, Kazarian K, Telkov M, Kaprelyants AS, Kell DB, Young M (2002a). The *rpf* gene of *Micrococcus luteus* encodes an essential secreted growth factor. Molecular Microbiology.

[ref-25] Mukamolova GV, Turapov OA, Young DI, Kaprelyants AS, Kell DB, Young M (2002b). A family of autocrine growth factors in *Mycobacterium tuberculosis*. Molecular Microbiology.

[ref-26] Nikitushkin VD, Demina GR, Kaprelyants AS (2011). Effect of secreted Rpf protein on intracellular contacts in *Micrococcus luteus* and *Mycobacterium smegmatis* cultures. Microbiology.

[ref-27] Nikitushkin VD, Demina GR, Kaprelyants AS (2016). Rpf proteins are the factors of reactivation of the dormant forms of actinobacteria. Biochemistry (Moscow).

[ref-28] Nikitushkin VD, Demina GR, Shleeva MO, Guryanova SV, Ruggiero A, Berisio R, Kaprelyants AS (2015). A product of RpfB and RipA joint enzymatic action promotes the resuscitation of dormant mycobacteria. FEBS Journal.

[ref-29] Oliver JD (2010). Recent findings on the viable but nonculturable state in pathogenic bacteria. FEMS Microbiology Reviews.

[ref-30] Ravagnani A, Finan CL, Young M (2005). A novel firmicute protein family related to the actinobacterial resuscitation-promoting factors by non-orthologous domain displacement. BMC Genomics.

[ref-31] Rittershaus ES, Baek SH, Sassetti CM (2013). The normalcy of dormancy: common themes in microbial quiescence. Cell Host & Microbe.

[ref-32] Rosser A, Stover C, Pareek M, Mukamolova GV (2017). Resuscitation-promoting factors are important determinants of the pathophysiology in *Mycobacterium tuberculosis* infection. Critical Reviews in Microbiology.

[ref-33] Sachidanandham R, Yew-Hoong Gin K (2009). A dormancy state in nonspore-forming bacteria. Applied Microbiology and Biotechnology.

[ref-34] Sexton DL, St-Onge RJ, Haiser HJ, Yousef MR, Brady L, Gao C, Leonard J, Elliot MA (2015). Resuscitation-promoting factors are cell wall-lytic enzymes with important roles in the germination and growth of *Streptomyces coelicolor*. Journal of Bacteriology.

[ref-35] Shleeva MO, Mukamolova GV, Telkov MV, Berezinskaya TL, Syroeshkin AV, Biketov SF, Kaprelyants AS (2003). Formation of non-culturable *Mycobacterium tuberculosis* and their regeneration. Mikrobiologiya.

[ref-36] Squeglia F, Ruggiero A, Romano M, Vitagliano L, Berisio R (2014). Mutational and structural study of RipA, a key enzyme in *Mycobacterium tuberculosis* cell division: evidence for the L-to-D inversion of configuration of the catalytic cysteine. Acta Crystallographica Section D Biological Crystallography.

[ref-37] Van der Geize R, Dijkhuize L (2004). Harnessing the catabolic diversity of *Rhodococci* for environmental and biotechnological applications. Current Opinion in Microbiology.

[ref-38] Voloshin SA, Kapreliants AS (2005). Cell aggregation in cultures of *Micrococcus luteus* studied by dynamic light scattering, Prikl Biokhim. Mikrobiologiya.

[ref-39] Yang Z, Chen JX, Qin B, Li YY, Zhang QF, Zhao X, Zhou T (2015). Characterization and catabolic gene detection of three oil-degrading *Rhodococcus* spp. Chinese Journal of Applied & Environmental Biology.

[ref-40] Yue L, Chen JX, Yang Z, Li YY, Rui WH, Wang YG (2018). Gene cloning and functional domains analysis of recovery promoting factors in *Rhodococcus erythropolis* rpf. Genomics and Applied Biology.

