# Supplementary material for: Enzyme characterization and biological activities of a resuscitation promoting factor from an oil degrading bacterium Rhodococcus erythropolis KB1
Source: PeerJ. 2019 May 21;7:e6951. doi: 10.7717/peerj.6951 (PMC6534110; doi:10.7717/peerj.6951)
Supplement: Supplemental Information 2 [file peerj-07-6951-s002.doc]

muralytic activity

| Name | Data | | | average value | U/mg |
| --- | --- | --- | --- | --- | --- |
| Rpf-1wide | 1.86817 | 1.80146 | 1.78097 | 1.760 | 1760 |
| E51A | 1.57159 | 1.79446 | 1.52099 | 1.573 | 1573 |
| C50G-C114T | 0.288384 | 0.294309 | 0.292999 | 0.234 | 234 |
| Q69K | 0.38402 | 0.294567 | 0.430458 | 0.245 | 245 |
| D45A-E51K | 0.492961 | 0.275423 | 0.223972 | 0.275 | 275 |
| W75V | 3.57405 | 3.45631 | 3.44289 | 3.435 | 3435 |
| T74A | 1.76875 | 1.75416 | 1.74352 | 1.699 | 1699 |
| T60A | 1.88195 | 1.72861 | 1.66176 | 1.701 | 1701 |

Protease activity

| Name | Data | | | average value | U/mg |
| --- | --- | --- | --- | --- | --- |
| Rpf-1wide | 0.066 | 0.066 | 0.066 | 0.007 | 1634 |
| E51A | 0.055 | 0.055 | 0.055 | / |  |
| C50G-C114T | 0.055 | 0.055 | 0.055 | / |  |
| Q69K | 0.060 | 0.059 | 0.059 | / |  |
| D45A-E51K | 0.052 | 0.052 | 0.052 | / |  |
| W75V | 0.054 | 0.054 | 0.054 | / |  |
| T74A | 0.055 | 0.056 | 0.055 | / |  |
| T60A | 0.071 | 0.072 | 0.071 | 0.012 | 1567 |
| Inactivated protein | 0.059 | 0.059 | 0.059 | - |  |
| Distilled water | 0.057 | 0.058 | 0.058 | - |  |
