# Supplementary material for: Enzyme characterization and biological activities of a resuscitation promoting factor from an oil degrading bacterium Rhodococcus erythropolis KB1
Source: PeerJ. 2019 May 21;7:e6951. doi: 10.7717/peerj.6951 (PMC6534110; doi:10.7717/peerj.6951)
Supplement: Supplemental Information 3 [file peerj-07-6951-s003.docx]

pET-32a（+）Protein enzyme activity

Muralytic activity：0

Protease activity：0

The growth ability of the *R. erythropolis* cells with addition of pET-32a（+）protein as shown in the table below,Closed to inactivated protein data.

| Incubation time | OD595nm |
| --- | --- |
| 0 | 0.031±0.003 |
| 4 | 0.039±0.002 |
| 8 | 0.049±0.003 |
| 12 | 0.078±0.004 |
| 24 | 0.179±0.011 |
| 36 | 0.406±0.016 |
| 48 | 1.051±0.023 |
| 60 | 2.121±0.019 |
| 72 | 3.017±0.021 |
| 84 | 2.999±0.047 |

Recovery of the *R. erythropolis* KB1 cells under cold and starved condition by addition of the pET-32a（+）protein culturable counts was 8.19±0.27 CFU mL^-1^ (×10^3^),Closed to inactivated protein data.
